# Use of dicarboxylic acids and polyphenols to attenuate reticular pH drop and acute phase response in dairy heifers fed a high grain diet

**DOI:** 10.1186/s12917-014-0277-5

**Published:** 2014-11-26

**Authors:** Roberta De Nardi, Giorgio Marchesini, Jan C Plaizier, Shucong Li, Ehsan Khafipour, Rebecca Ricci, Igino Andrighetto, Severino Segato

**Affiliations:** Department of Animal Medicine Production and Health, University of Padova, Legnaro, (PD) 35020 Italy; Department of Animal Science, University of Manitoba, Winnipeg, MB Canada

**Keywords:** Reticular pH, Acute phase protein, Fumarate-malate, Polyphenol, High grain diet, Heifer

## Abstract

**Background:**

The aim of this study was to determine the ability of two feed additives, a fumarate-malate (FM) and a polyphenol-essential oil mixture (PM), in attenuating the drop of ruminal pH and the metabolic and immune response resulting from an excessively high grain diet. Six heifers were used in a 3 × 3 Latin square experiment and fed a low starch (LS) diet for 14 d, followed by a high starch (HS) diet for 8 d (NDF 33.6%, starch 30.0% DM). In the last 5 days of each period, barley meal was added to decrease rumen pH. During HS feeding all animals were randomly assigned to one of the following three dietary treatments: no supplement/control (CT), a daily dose of 60 g/d of FM, or 100 g/d of PM. Reticular pH was continuously recorded using wireless boluses. On d 21 of each period, rumen fluid was collected by rumenocentesis (1400 h), together with blood (0800 h) and fecal samples (0800, 1400, and 2100 h).

**Results:**

The correlation coefficient of pH values obtained using the boluses and rumenocentesis was 0.83. Compared with CT and PM, the FM treatment led to a lower DMI. Nadir pH was lowest during CT (5.40, 5.69, and 5.62 for CT, FM and PM, respectively), confirming the effectiveness of both supplements in reducing the pH drop caused by high grain feeding. This result was confirmed by the highest average time spent daily below 5.6 pH (199, 16 and 18 min/d) and by the highest acetate to propionate ratio of the CT fed heifers. The PM decreased the concentrations of neutrophils (2.9, 3.2, and 2.8 10^9^/L) and acute phase proteins: SAA (37.1, 28.6 and 20.1 μg/mL), LBP (4.1, 3.8, and 2.9 μg/mL), and Hp (675, 695 and 601 μg/mL). Free lipopolysaccharides (LPS) were detected in blood and feces, but their concentrations were not affected by treatments, as the remaining blood variables.

**Conclusions:**

Data suggest that both additives could be useful in attenuating the effects of excessive grain feeding on rumen pH, but the PM supplement was more effective than FM in reducing the inflammatory response compared to CT.

## Background

In the grain-based and energy-dense diets typically fed to highly productive dairy cows, the rapid ruminal fermentation of starch and sugars can lead to an accumulation of volatile fatty acids (VFA) and/or lactate, which causes a drop of pH and a shift in the balance of rumen microorganisms [1,2]. Especially, when switching from a low-energy high-forage diet to a high-energy early lactation diet, the rate of microbial VFA production exceeds the rate at which they can be cleared from the rumen [3,4]. As a result, VFA accumulates and the rumen pH declines [5]. The condition in which pH is depressed for prolonged periods each day is defined as subacute ruminal acidosis (SARA). This is an ongoing and costly digestive disorder of dairy cows [6,7], affecting rumen fermentation, production and immune response [8-10]. Good management and nutritional strategies alone [8,5] are not always enough to avoid the onset of SARA in high yielding dairy cows and for this reason other preventative measures have been suggested. This included physical manipulation of fiber and grain particle size [8,11,12], the inclusion of antibiotics in the diet [13], the use of yeasts and probiotic bacteria [14,15], and the addition of dicarboxylic acids [16], flavonoids [17] or essential oils [18] to manipulate rumen microbial communities and subsequently ruminal fermentation.

The dicarboxylic acids malate and fumarate have been proposed as modifiers of ruminal fermentation and as an alternative to antibiotics [19,16]. It has been suggested that these acids may increase the activity of the succinate-propionate metabolic pathway in several rumen bacteria which results in increased lactic acid uptake and production of propionate [2,20]. Flavonoids, essential oils and other plant-derived compounds have been proposed as feed supplements for their anti-inflammatory, antioxidant, and antimicrobial properties [21,18,22]. In addition, flavonoids have been reported to be effective in preventing pH reduction through modifying the activity of lactating-consuming bacteria and promoting the growth of propionate-producing bacteria [17]. Moreover, essential oils are known to have antimicrobial properties, and have been suggested to act as rumen fermentation modifiers [18].

The aim of this study was to determine the effects of two feed additives, a fumarate-malate mixture and a polyphenol-essential oil mixture, in attenuating the drop of rumen pH and the changes in metabolites and inflammatory markers in blood and rumen fluid due to feeding high grain diets to heifers.

## Methods

### Animals and experimental design

The experimental protocol and all the procedures used in this study were approved by the Animal Ethics Committee of the University of Padova, Italy (CEASA, approval number 73/2012).

Six purebred Italian Holstein-Friesian non-pregnant heifers with an average body weight (BW) of 556 ± 33 kg (mean ± SD) were used in a 3 × 3 Latin square design. Heifers were born in a dairy farm located in the lowland of Vicenza (Veneto Region, Italy) where the experimental trial took place. After the experiment, heifers were inseminated to begin their normal productive life as dairy cows. Animals were randomly assigned to one of the three dietary treatments during each period. Each period lasted 22 d: 14 d of an adjustment phase followed by 8 d of data collection. Heifers were kept in individual pens in loose housing conditions and had unlimited access to fresh water. All of the heifers were examined by a veterinarian to evaluate their health status throughout the trial.

### Dietary treatments

During each period heifers were fed a low starch (LS) diet for 14 d *ad libitum*, followed by a high starch (HS) diet for 8 d: from d 15 to d 22 (Table 1). Diets were provided as TMR once daily at 0800 h. During the HS feeding heifers were offered one of three dietary treatments: i) no supplement, CT treatment; ii) a daily dose of 60 g of fumarate-malate mixture (RumenStabiliser®, DR. Eckel, Niederzissen, Germany), FM treatment; iii) a daily dose of 100 g of polyphenol-essential oil mixture (Anta®Phyt RU, DR. Eckel, Niederzissen, Germany), PM treatment.

**Table 1:** **Ingredients and proximate composition of diets fed to heifers**

	**Diet** ^**1**^	
**Item**	**LS**	**HS**
Ingredients, % of DM		
Corn meal (0.5 mm)	23.0	34.0
Hay	24.0	22.0
Dehydrated alfalfa hay	18.0	16.0
Extruded soybean hull	7.0	2.0
Barley meal	7.0	9.0
Straw	6.5	5.5
Molasses	2.0	2.0
Sugar beet dry pulps	4.0	1.0
Corn gluten meal	6.0	2.0
Sunflower	-	2.0
Soybean meal	-	2.0
Vitamin and mineral mix	1.5	1.5
Extruded de-hulled soybean	1.0	1.0
Proximate composition		
DM, %	88.9	88.8
Crude protein, % of DM	12.5	12.5
Crude fat, % of DM	3.5	3.3
NDF, % of DM	39.8	33.6
Crude ash, % of DM	7.8	7.4
ADF, % of DM	21.4	19.2
NFC, ^2^% of DM	36.4	43.2
Starch, % of DM	24.0	30.0

^1^Diets: LS = low starch; HS = high starch.

^2^NFC = 100 – (NDF + crude protein + crude fat + crude ash).

The FM supplement is an organic acid and buffer blend made of magnesium fumarate, malic acid, sodium acetate and sodium bicarbonate, whereas PM is a blend made of natural plant extracts, characterised by a high content of phenolic compounds comprising mostly flavonoids (1.88 mg/g). The amount of dicarboxylic acids and flavonoids reported to be effective in the modification of ruminal fermentations in the literature [23,24,17] was taken into account in determining the supplement doses. The two supplements were given together with approximately 1 kg of TMR and their complete intake was verified by an operator before distributing the rest of the ration.

With the aim to induce a drop in rumen pH below 5.6 for more than 3 h/d, the threshold pH established for SARA [6], from the d 18 to the d 22, barley meal (32.5% NDF and 53.1% starch on DM) was top dressed on the TMR. The quantity of barley meal was gradually increased from 0.5 to 1.5 kg (with 250 g increment *per* day) to prevent from a fast drop of pH that could have led to acute ruminal acidosis. The barley meal provision and the control of its intake were performed using the same technique described for the supplements in order to guarantee the same level of intake throughout the treatments.

### Feed intake and feed analyses

The weight of the feed offered and refused was recorded daily during the HS feeding. Samples of both the LS and HS diets were collected twice for each period and analyzed for proximate composition (Table 1). Feed samples were dried at 60°C for 48 h and ground to pass through a 1-mm screen and then analysed for DM, crude protein (CP), crude fat and crude ash according to AOAC [25], whilst NDF and ADF were analyzed according to Van Soest et al. [26] using α-amylase and a Fibre Analyser (ANKOM/ 2000; ANKOM Technology, New York, NY, USA). The starch content was determined using high-performance liquid chromatography equipped with a LC 9A pump, SIL auto sampler and the RID-10 A model (Shimadzu, Tokyo, Japan); separations were achieved using a 300 × 7.8 mm Aminex HPX-87H column and one pre-column (Micro Guard Cation H 30 × 4.6 mm, Bio-Rad, Hercules, CA, USA) at 40°C [27].

### Sampling and analysis of ruminal fluid and reticular pH

The pH of the reticulum was continuously measured during the entire trial using wireless boluses (SX-1042, SmaXtec Animal Care GmbH, Graz, Austria). The boluses were calibrated and delivered orally in the reticulum using a balling gun and their positions were verified using ultrasound measurement. The pH readings were recorded every 10 min [28].

The pH data of the last two days of each period (d 21 and 22), measured by the bolus in each animal, were summarized daily as the average, maximum and nadir pH. With the purpose to make comparison with data present in literature, the amounts of time *per* day that the pH was below three ruminal pH thresholds (pH <5.6, pH <5.8 and pH <6.3) were determined for each heifer during the three experimental periods, as described by Gozho et al. [29].

In this study, the pH threshold values were selected because the duration of the rumen pH below 5.6 is related to an increase of the intensity of the inflammatory acute phase response [30]; pH <5.8 is harmful to ruminal cellulolytic bacteria [31]; pH <6.3 is proposed by Sato et al. [32] for SARA determination from reticular fluid, given that the reticular pH is higher than the ruminal pH, due to mixing and dilution with saliva [33].

Rumenocentesis was performed at 1400 h (6 hours after TMR distribution) on d 21 of each period using a 13G 105-mm needle [34]. The pH of ruminal fluid was measured immediately using a portable pH meter (Piccolo, Hanna Instruments, Villafranca Padovana, Italy) and compared to the average pH values recorded by the boluses just prior to and immediately after the rumenocentesis time [7]. Rumen fluid samples were strained through 4 layers of sterile cheesecloth and were collected and divided into 2 portions. The first portion of each sample was transferred into a 50-mL sterile tube and kept on ice until the processing required for lipopolysaccharide (LPS) determination, as described by Li et al. [9]. For LPS, rumen fluid samples were centrifuged at 12,000 × *g* for 40 min at 4°C; the supernatant was aspirated, filtered using 0.22-μm sterile, pyrogen free filter (Millex, Millipore Corporation, Bedford, MA, USA) and collected into depyrogenated glass tubes (heated at 200°C for 2.5 h). The samples were heated at 100°C for 30 min, cooled at room temperature for 10 min and stored at −20°C until analysis. Free LPS in rumen fluid was measured by a chromogenic kinetic *Limulus* amebocyte lysate (LAL) assay (50–650U, Kinetic-QCL, Lonza Group Ltd., Basel, Switzerland) in a 96-well microplate using an incubating microplate spectrophotometer (Synergy H4 Hybrid Multi-Mode Microplate Reader, Bio-Tek, Instruments, Inc., Winooski, VT, USA). Rumen fluid samples were diluted 1:67,100 using LAL water pyrogen-free (LAL Reagent Water, Lonza Group Ltd., Basel, Switzerland), with the final dilution being made of 50% diluted sample and 50% β-glucan blocker (N.190, Lonza, Walkersville, MD, USA).

The second portion of each rumen fluid sample (5 mL) was centrifuged at 3,000 × *g* for 15 min at 4°C, for VFA, lactate and ammonia N analyses. Samples were acidified with 0.6 M HCl to inhibit microbial activity and minimize volatilization (dilution 5:1) and stored at −20°C until analysis.

After thawing, samples were centrifuged at 4,000 × *g* for 30 min at 4°C and the supernatants were filtered using 0.45-μm Phenex-RC filters (Phenomenex, Castel Maggiore, Italy). One subsample of the filtrate was analysed for ammonia N using a SmartChem 200 spectrophotometer (Unity Scientific, Brookfield, CT, USA). For the VFA and lactate analysis, a second subsample was injected into an HPLC system complete with an LC 9A Shimadzu pump, a SIL 10A auto sampler and a RID-model Shimadzu 10A detector (Shimadzu, Tokyo, Japan). Analytes separation was performed at 40°C using an Aminex HPX-87H column (300 × 7.8 mm) and one pre-column (Bio-Rad, Hercules, CA, USA). Class VP software was used for data collection and integration. For the complete HPLC analysis, a 30 min isocratic program was run with 0.025 N H_2_SO_4_ as the mobile phase at a flow rate of 0.6 mL/min. Peaks of analytes were identified by comparing the retention times of standard mixtures to those of the samples and quantification was based on peak area measurements that were compared with that of an external standard.

### Blood collection and analysis

Blood samples (20 mL) from the jugular vein were collected from each animal before the feed delivery at 0800 h on d 21 of each period into lithium-heparin, K3 EDTA and tubes without anticoagulant (Vacuette, Greiner Bio-One, Kremsmuenster, Austria). The blood from the K3 EDTA tubes and one subsample of lithium-heparin-preserved blood were refrigerated (4°C) and analyzed within 1 h for a complete blood cell count and blood gas analysis, respectively. The other subsamples were immediately centrifuged at 1,500 × *g* for 15 min at 4°C for plasma and serum separation, and were preserved at –80°C until analysis.

Complete blood cell count with leukocyte formula was performed using an automated cell counter (Cell Dyn 3500, Abbott Laboratories, Abbott Park, IL, USA). Blood gas analysis was performed within 1 h from the collection in a calibrated blood gas analyzer (Synthesis 15, IL Instrumentation Laboratory SpA, Milano, Italy) and blood pH, partial pressure of carbon dioxide, partial pressure of oxygen, and the percentage of oxyhemoglobin and reduced hemoglobin were determined. The bicarbonate levels and measured oxygen saturation were calculated. Measurements were performed as recommended by the National Committee of Blood Laboratory Standards [35]. The plasma was analyzed for the hematological profile: glucose, cholesterol (CHOL), non-esterified fatty acids (NEFA), β-hydroxybutyrate (β-HB), aspartate aminotransferase (AST), γ-glutamyl transferase (γGT) by using a Roche Cobas C501 automatic analyzer (Roche Diagnostics, Indianapolis, IN, USA). Plasma was also analyzed for LPS, interleukin 6 (IL-6) cytokine and acute phase proteins: serum amyloid A (SAA), LPS binding protein (LBP) and haptoglobin (Hp). The SAA, LBP and Hp were measured using the following commercially available ELISA kits, respectively, as described by Gozho et al. [36] and Khafipour et al. [37]: TP802-2 (Tridelta Diagnostics Ltd., Maynooth, Co. Kildare, Ireland), HK503 (Hycult Biotech Inc., Plymouth Meeting, PA, USA), TP801-Mk2 (Tridelta Diagnostics Ltd., Maynooth, Co. Kildare, Ireland) and ESS0029 (Thermo Scientific, Rockford, IL, USA). A chromogenic LAL assay (Kinetic-QCL™, Lonza Group Ltd., Basel, Switzerland) was used to measure the concentration of LPS in plasma as described by Khafipour et al. [37] and Li et al. [9]. Samples were diluted 1:4 with LAL Reagent Water. Samples were incubated at 70°C for 30 min. Heated samples (100 μL) were added to a 1:7 diluted 10 mM MgCl_2_ solution (Lonza Group Ltd., Basel, Switzerland) and Pyrosperse® (N188, Lonza Group Ltd., Basel, Switzerland).

### Fecal sampling and analyses

Fecal samples were collected from the rectum at 0800, 1400 and 2100 h on the d 21 of each period. The pH was measured immediately using a portable pH meter (Piccolo, Hanna Instruments, Villafranca Padovana, Italy). About 40 g sample was processed for LPS analyses using the same procedure adopted for rumen fluid, but with a dilution of 1:26,000 [38]. These samples were stored at −20°C until analysis.

### Statistical analysis

The DMI and reticular pH data were analyzed using the MIXED procedure with a compound symmetry structure. The linear model considered the fixed effect of dietary treatment (CT, FM and PM), period, day (repeated measure) and their interactions. Heifer was included as random effect. Ruminal and blood data were analyzed according to the same model but without the day effect (data recorded on d 21). Moreover, feces data were also analyzed according to the described model, but the day was replaced by daily sampling time effect (0800, 1400 and 2100 h). If significant treatment effects were detected (*P* <0.05), the LS*means* were compared using the probability of differences (PDIFF) option and the Tukey adjustment test. To obtain a normal distribution and homogeneous residual error, ruminal LPS, blood LPS and IL-6 data were log transformed. Means and confidence intervals were then reported in tables after antilog transformation.

The average amount of time for each heifer with a reticular pH below the three established pH thresholds (<5.6, <5.8 and <6.3) were not normally distributed even after transformation. Thus, these data were tested using the non-parametric Kruskal-Wallis criteria using the Dunn’s multiple pairwise comparisons. Pearson’s correlation coefficient (PROC CORR) was assessed between the reticular and ruminal pH measurements.

All of the statistical analyses were performed using SAS software (2010, release 9.3; SAS Institute Inc., Cary, NC, USA). The effects were considered significant at *P* <0.05 and trends were discussed at 0.05 < *P* <0.10.

## Results

### Weight gain and DMI

At the end of the trial, the heifers weighed an average of 625 ± 37 kg and the average daily gain was 1.04 ± 0.09 kg/d. The DMI was affected by the treatment (*P* =0.021) and was the lowest on the FM diet (Table 2). Moreover the DMI was affected by day and was found to increase from the 18^th^ (14.2 kg/d) to the 21^st^ d (15.1 kg/d) and then slightly decrease on the 22^nd^ d (14.7 kg/d).

**Table 2:** **Effect of dietary treatment on DMI, reticular pH, daily average of time spent below the three reticular pH thresholds, ruminal volatile fatty acid (VFA) N-NH3 and lipopolysaccharides (LPS)**

	**Treatment** ^**1**^			**SEM**	***P*** **-value**
**Item**	**CT**	**FM**	**PM**		
DMI, ^2^ kg/d	14.5^a^	13.4^b^	14.7^a^	0.62	0.021
Reticular pH					
Average	6.04	6.11	6.09	0.067	0.466
Maximum	6.61	6.54	6.55	0.063	0.569
Nadir	5.40^b^	5.69^a^	5.62^ab^	0.106	0.037
pH <5.6, ^3^ min	199^a^	16^b^	18^b^	-	0.022
pH <5.8, ^3^ min	360	190	171	-	0.311
pH <6.3, min	1156	1118	1071	86.4	0.546
VFA, mM					
Acetate	59.2	60.9	60.6	3.60	0.911
Propionate	26.5	30.8	27.9	2.07	0.249
Butyrate	10.5	10.7	9.8	0.69	0.683
Ac:Pr^4^	2.05^α^	1.66^β^	1.77^αβ^	0.125	0.048
N-NH_3_, mg/dL	50.1	54.5	31.1	9.70	0.303
LPS^5, 6^, × 10^3^ EU/mL	15.7	6.0	8.9	(5.3–16.7)	0.172

^1^Treatments: control diet (CT); fumarate-malate mixture (FM); polyphenol-essential oil mixture (PM).

^2^For DMI the repeated effect of day was significant (*P* <0.001).

^3^ *P*-values and superscript letters (^a, b^: *P* <0.05) are given by using the non-parametric Kruskal–Wallis test and the Dunn’s multiple pairwise comparison.

^4^Acetate to propionate ratio.

^5^Statistical analysis was conducted on natural logarithm (ln) transformed data that are presented as ln back transformed and 95%-confidence interval in brackets.

^6^LPS were reported as endotoxin unit.

^a, b^Means with different superscripts within a row differ (*P* <0.05).

^α, β^Means with different superscripts within a row differ (*P* <0.10).

### pH, VFA, N-NH_3_ and free LPS in rumen fluid

The correlation coefficient (r) between the pH values obtained using the reticular boluses and rumenocentesis was 0.83 (*P* <0.001). The dietary treatment significantly affected nadir pH, the acetate to propionate ratio and the time spent below pH 5.6, but not the concentrations of VFA, ammonia N, LPS, mean and maximum pH and the time spent below pH 5.8 and 6.3 (Table 2). The FM diet led to the highest nadir pH (*P* = 0.037), whereas both FM and PM significantly reduced the time spent below 5.6 compared to CT (199 vs. 16 vs. 18 min/d, for CT, FM and PM respectively; *P* = 0.022). The acetate to propionate ratio (Table 2) was the highest with CT and the lowest with FM (*P* = 0.048). The concentration of lactate was almost negligible (<0.01 mM) and was not affected by treatment.

### Blood variables

Peripheral blood concentrations of acute phase proteins (SAA, LBP and Hp) were affected by treatment, whereas concentrations of IL-6 and LPS, total blood cell count, blood gas and haematological profile variables did not show any differences among treatments. An exception was neutrophils (NEU) that were significantly lower (P = 0.084) in the PM treatment (Tables 3 and 4). The PM treatment decreased the concentration of SAA (*P* = 0.036), LBP (*P* = 0.048) and Hp (*P* = 0.084), whereas FM showed intermediate values between PM and CT for SAA and LBP.

**Table 3:** **Effects of dietary treatment on concentrations of serum amyloid A (SAA), lipopolysaccharide binding protein (LBP), haptoglobin (Hp), interleukin-6 (IL-6), blood lipopolysaccharides (LPS), and fecal pH and LPS**

	**Treatment** ^**1**^			**SEM**	***P*** **-value**
**Item**	**CT**	**FM**	**PM**		
Blood					
SAA, μg/mL	37.1^a^	28.6^ab^	20.1^b^	5.21	0.036
LBP, μg/mL	4.1^a^	3.8^ab^	2.9^b^	0.65	0.048
Hp, μg/mL	675^αβ^	695^α^	601^β^	42.9	0.084
IL-6, ^2^ ng/mL	0.90	1.23	0.83	(0.0–4.1)	0.260
LPS, ^3^ EU/mL	0.35	0.36	0.42	0.082	0.387
Feces^4^					
pH	6.60	6.56	6.65	0.057	0.697
LPS, ^2,3^ × 10^3^ EU/mL	10.9	5.4	10.9	(5.4–13.9)	0.168

^1^Treatments: control diet (CT); fumarate-malate mixture (FM); polyphenol-essential oil mixture (PM).

^2^Statistical analysis was conducted on natural logarithm (ln) transformed data that are presented as ln back transformed and 95%-confidence interval in brackets.

^3^LPS were reported as endotoxin unit.

^4^For feces variables, the statistical model included also the repeated effect of daily sampling time (3 levels: 0800 vs. 1400 vs. 2100). The effect was significant for pH (*P* = 0.042), but not for LPS (*P* = 0.128).

^a, b^Means with different superscripts within a row differ (*P* <0.05).

^α, β^Means with different superscripts within a row differ (*P* <0.10).

**Table 4:** **Effects of dietary treatment on blood pH, count, gas and haematological profile**

	**Treatment** ^**1**^			**SEM**	***P*** **-value**
**Item** ^**2**^	**CT**	**FM**	**PM**		
pH	7.42	7.42	7.41	0.009	0.815
Red blood cells, 10^12^/L	6.6	6.5	6.6	0.27	0.441
White blood cells, 10^9^/L	8.2	8.4	8.1	0.24	0.726
Neutrophils, 10^9^/L	2.9^αβ^	3.2^α^	2.8^β^	0.27	0.084
Lymphocytes, 10^9^/L	4.1	3.9	3.9	0.19	0.835
Monocytes, 10^9^/L	0.82	0.87	0.95	0138	0.148
Basophils, 10^9^/L	0.10	0.09	0.10	0.021	0.794
Eosinophils, 10^9^/L	0.31	0.35	0.33	0.102	0.908
Hemoglobin, g/dL	11.4	11.2	11.5	0.26	0.128
Hematocrit, %	32.2	31.8	32.4	0.64	0.318
Platelets, K/μL	243	280	241	44.8	0.359
pCO_2_, mmHg	48.0	48.9	47.1	1.36	0.538
pO_2_, mmHg	60.2	71.1	59.8	5.66	0.299
HCO_3_^-^, mmol/L	31.2	32.0	30.5	0.62	0.235
Oxyhemoglobin, %	90.5	91.4	89.0	2.13	0.701
Reduced hemoglobin, %	6.8	6.0	8.4	2.12	0.693
sO_2_m, %	93.1	94.1	91.4	2.19	0.681
Glucose, mmol/L	4.45	4.55	4.50	0.137	0.818
Cholesterol, mmol/L	3.3	3.4	3.3	0.24	0.957
NEFA, meq/L	0.07	0.57	0.07	0.403	0.637
β-HB, mmol/L	0.33	0.29	0.41	0.049	0.325

^1^Treatments: control diet (CT); fumarate-malate mixture (FM); polyphenol-essential oil mixture (PM).

^2^pCO_2_, partial pressure of carbon dioxide; pO_2_, partial pressure of oxygen; HCO_3_^-^, bicarbonate level; sO_2m_, measured oxygen saturation; NEFA, non-esterified fatty acids; β-HB, β-hydroxybutyrate.

^α, β^Means with different superscripts within a row differ (*P* <0.10).

### Fecal variables

The pH and LPS concentration of feces were not affected by dietary treatment (Table 3), whereas fecal pH values were affected by time (*P* =0.042) and were 6.38, 6.71 and 6.69 at 0800, 1400 and 2100 h, respectively.

## Discussion

Our objective was to test if the addition of a supplement based on fumarate-malate (FM) or a polyphenol-essential oil mixture (PM) to an high grain and potentially subacute rumen acidosis-inducing diet alters ruminal fermentations and attenuates the reticular pH drop, the production of LPS and their consequences on the immune response and on biochemical and blood gas profiles that result from feeding this diet. For this purpose heifers were preferred to dairy cows to avoid the carry-over effects from the feeding on high grain diets in previous lactations. Both the addition of FM and PM reduced the drop of reticular nadir pH and the daily average time spent by the heifers below pH 5.6 when compared to CT diet. The heifers fed the latter diet spent 199 min below pH 5.6, which is more than the threshold for SARA proposed by Gozho et al. [30], even though the pH in the reticulum is reported to be higher than in the rumen [32,33]. Treatment did not affect the fecal pH, which confirms that ruminal pH is not closely related to the latter [8].

The concentration of LPS found in the rumen of CT-fed animals (raw data, 38,300 EU/mL) was similar to that reported by other authors after an episode of SARA induced by feeding high grain diets [30,39]. This means that the high grain diet led to a drop of rumen pH sufficient to trigger the production and the accumulation of LPS in rumen fluid. Although in other studies [37,9] high grain diets led to concentrations of ruminal LPS higher than that found in the present trial, it must be remembered that previous studies used cows instead of heifers. Heifers have lower feed intakes and are also subjected to less nutritional and metabolic stresses compared to cows, which may explain the relatively lower rumen LPS concentrations in our study.

The LPS are bacterial endotoxins which, when the mucosa of the digestive tract is damaged, can translocate into the bloodstream [40] and induce systemic immune response and metabolic alterations which can compromise animal health and performance [41].

The FM and PM treatments reduced the time spent below pH 5.6 to the same extent, but this effect, for FM-fed heifers, could be partially due to the reduction in DMI that was 7.5% lower than that found for CT and PM diets. This reduction is in agreement with that found by other authors [42,24] after the administration of dicarboxylic acids, although the dietary inclusion rate of the fumarate-malate based supplement in our study was much lower than that reported to cause DMI reduction [24].

The DMI was also affected by day, increasing from d 18 to d 21 and then decreasing on d 22. This effect is probably caused by the preference for barley meal whose amount was raised gradually along the last five days of each period. The reduction of DMI in d 22 corresponded to the drop of reticular pH at this time.

According to the literature [17,20,43] the mechanisms of action through which both fumarate-malate and polyphenols should reduce the drop of rumen pH are related to a change in the fermentation pattern and to the increasing of lactate utilization by some anaerobic lactate-consuming bacteria.

Martin [20] reported that fumarate and malate are intermediates of the citric acid cycle and that they may provide an electron sink for H_2_ that allows for increased lactate utilization by strictly anaerobic bacteria which use the *succinate-propionate pathway* to synthesize succinate and (or) propionate. Other authors [17] found that flavonoids modify the activity of some lactate-consuming bacteria, which rapidly metabolize lactate to VFA, thereby, preventing lactate accumulation in the rumen. However, in this study, the concentration of lactic acid was found to be negligible. This probably means that lactic acid was produced and immediately converted into VFA, as suggested by some authors, thereby preventing any accumulation of lactate in the rumen [43,44].

The action of both supplements in the rumen microbial fermentation pattern is also supported by the reduction in the acetate to propionate ratio by FM and PM compared to CT. In the rumen, the ratio between different products of carbohydrate fermentation depends on the hydrogen concentration, due to microbial interspecies hydrogen transfer [45]. The possible effect of some additives on hydrogen -producing and hydrogen -consuming microorganisms, can lead the pyruvate to be converted to different VFA, CO_2_, hydrogen and intermediary products, according to a fermentation pattern that varies with microbial species [45].

The PM treatment resulted in the greater attenuation of the increase in the concentrations of APP (SAA, LBP and Hp) in blood after high grain feeding. The increase of APP, plasma proteins produced mainly from the liver and used as sensitive markers of the inflammation, is the expression of a systemic and innate reaction of the organism to inflammation triggered by external (pathogens, toxins, etc.) or internal (tissue damage, etc.) stimuli [46]. Many APP, including SAA and Hp, are poorly specific for pathogens and toxins [47]. However, in our study their increase is likely due to the translocation of LPS out of the digestive tract into the portal circulation [36,37,48]. As routinely checks by a veterinarian excluded other possible causes of inflammation, like laminitis or respiratory disorders.

The SAA has different functions but mainly modulates innate immune reactions and in particular the migration of monocytes and neutrophils, whereas Hp has anti-inflammatory properties; LBP is triggered by bacterial infections and helps in the neutralization of LPS and in the activation of a cascade of reactions that leads to the release of cytokines, among which IL-6, that are necessary for the activation of the immune system [10,46,47]. The FM showed intermediate values among treatments for the concentrations of SAA and LBP, but the highest value for Hp, proving that its effect in attenuating the inflammation process, due to the rumen pH drop, is lower than that of PM. The concentration of LBP was lower than that found by other authors after high grain feeding [39,48]. This could be possibly related to the fact that heifers are not subjected to as many chronic disorders, and nutritional and metabolic stresses, that could may have occurred in cows during previous lactations, and thereby affect the concentrations of LBP [46].

The lack of differences among treatments in LPS and IL-6 blood concentrations suggests that these variables are less sensitive to the systemic effects of rumen pH drop than APP. LPS translocated from the digestive tract to portal blood are subject to a high clearance rate in the liver [9], which resulted in the drop of LPS concentration in the peripheral blood and likely in the reduction of possible differences.

The effect of PM in APP concentrations, especially SAA, is in agreement with its low concentration in neutrophils, since SAA influences the release and function of neutrophils during the acute phase response. The remaining blood variables were not affected by treatment, mainly because all the heifers were fed on the same high grain diet.

## Conclusions

Both additives were successful in attenuating the reticular pH drop (time spent <5.6) compared to control in heifers fed a high grain based diet. Moreover the polyphenol based supplement was effective in limiting the acute phase response without interfering with DMI. Rumen, blood and feces LPS concentrations were not affected by dietary treatment probably due to wide variability and the mechanism of translocation and/or clearance in the liver. Further studies are needed to better understand the influence of tested compounds on the rumen microbial community and on dairy cow performance.
